# Partial role of volatile organic compounds in behavioural responses of mice to bedding from cancer-affected congeners

**DOI:** 10.1242/bio.060324

**Published:** 2024-10-01

**Authors:** Flora Gouzerh, Laurent Dormont, Bruno Buatois, Maxime R. Hervé, Maicol Mancini, Antonio Maraver, Frédéric Thomas, Guila Ganem

**Affiliations:** ^1^ CREEC/ MIVEGEC, Centre de Recherches Ecologiques et Evolutives sur le Cancer/Maladies infectieuses et Vecteurs: Ecologie, Génétique, Evolution et Contrôle, UMR IRD 224-CNRS 5290-University of Montpellier, Montpellier, France; ^2^ CEFE, Centre d’écologie fonctionnelle et évolutive, Université Montpellier, CNRS, EPHE, IRD, University of Paul Valery Montpellier 3, Montpellier, France; ^3^ IGEPP, Institut de génétique, environnement et protection des plantes, INRAE, Institut Agro, University of Rennes, Rennes, France; ^4^ IRCM, Institut de recherche en cancérologie de Montpellier, Inserm U1194-ICM-Université Montpellier, Montpellier, France; ^5^ ISEM, Univ Montpellier, CNRS, IRD, Montpellier, France

**Keywords:** SPME, Odour signature, Soiled bedding, Cancer biomarkers, Inter-SPME-fibre reliability, EGFR cancer, *Mus musculus*

## Abstract

Tumours induce changes in body odours. We compared volatile organic compounds (VOCs) in soiled bedding of a lung adenocarcinoma male mouse model in which cancer had (CC) versus had not (NC) been induced by doxycycline at three conditions: before (T0), after 2 weeks (T2; early tumour development), after 12 weeks (T12; late tumour development) of the induction. In an earlier study, wild-derived mice behaviourally discriminated between CC and NC soiled bedding at T2 and T12. Here, we sought to identify VOCs present in the same soiled bedding that could have triggered the behavioural discrimination. Solid phase micro-extraction was performed to extract VOCs from 3 g-sample stimuli. While wild-derived mice could discriminate the odour of cancerous mice at a very early stage of tumour development (T2), the present study did not identify VOCs that could explain this behaviour. However, consistent with the earlier behavioural study, four VOCs, including two well-known male mouse sex pheromones, were found to be present in significantly different proportions in soiled bedding of CC as compared to NC at T12. We discuss the potential involvement of non-volatile molecules such as proteins and peptides in behavioural discrimination of early tumour development (T2), and point-out VOCs that could help diagnose cancer.

## Teaser

**Summary:** We sought to identify odorant molecules that could have triggered behavioural discrimination of cancerous mice, we show that volatile organic compounds may only partly be involved in mice cancer discrimination.

## INTRODUCTION

Volatile organic compounds (VOCs) are small carbon-based molecules that are characterised by their volatility at ambient temperatures. Over the last few decades, increasing attention has been devoted to exploring the relevance of VOCs emitted by the body as potential biomarkers of pathologies. VOCs are emitted and can be analysed from the breath, skin, saliva, sweat, blood, urine, and faeces. They are also commonly used as biomarkers of various diseases (Sethi et al., 2013; Shirasu and Touhara, 2011) such as fibrosis (Barker et al., 2006; Jalali et al., 2016), asthma (Ibrahim et al., 2011), Alzheimer's disease (Mazzatenta et al., 2015), diabetes, and tuberculosis (Phillips et al., 2004, 2007). Importantly, VOCs have received increasing attention in relation to the early detection of cancers (Gouzerh et al., 2022b). Such compounds, considered to be potential markers of differences between healthy and cancerous humans were identified in different odour sources – exhaled air, urine, blood, and faeces (Gouzerh et al., 2022b).

Previous studies on various diseases, and in particular cancer, have highlighted the ability of some animals, such as hamsters, fish or monkeys, to detect their sick congeners thanks to their olfactory capacity (Clayton, 1991; Gouzerh et al., 2022a; Kavaliers et al., 2020; Kennedy et al., 1987; Kokocinska-Kusiak et al., 2020; O'Shea et al., 2002; Poirotte et al., 2017; Sato et al., 2017). Mice have a highly developed sense of smell, and are able to detect odour signals and to discriminate between healthy and diseased conspecifics (Ehman and Scott, 2001; Kavaliers and Colwell, 1995; Kavaliers et al., 2005) or the status of an individual (Brennan, 2009; Chamero et al., 2007; Li et al., 2013; Restrepo et al., 2006). A large number of VOCs are released from mice urine. Among these VOCs are male sex pheromones such as brevicomine and thiazoline, which induce female puberty acceleration and oestrus (Wyatt, 2003), and urinary levels were found to differ between cancerous and non-cancerous mice. Kokocinska-Kusiak et al. (2020) identified that VOCs such as hexane and methylene chloride decreased in mice with melanoma compared to non-cancerous mice, while other compounds such as cetone, 1-methyl-6,7-dioxabicyclo[3.2.1]octane, and nitromethane, were found only in the urine of mice with melanoma. To the best of our knowledge, apart from Kokocinska-Kusiak et al. (2020) and Matsumura et al. (2010), no other studies using animals' noses to detect cancer, attempted to identify the odorant molecules potentially involved in this behavioural discrimination.

In an earlier study we reported that wild-derived mice exposed to the soiled bedding of the CCSP-rtTA / EGFRT790 M/L858R mouse model (i.e. mice that develop lung adenocarcinoma following doxycycline induction) could discriminate between soiled bedding of non-cancerous versus cancerous mice both at an early and at a late stage of tumour development (Gouzerh et al., 2022a). Here we investigated whether the composition of VOCs varied in the soiled bedding of the two types of mice, in order to identify candidate molecules that could be involved in the behavioural discrimination evidenced in our earlier study. To this aim, we analysed the VOC composition in the samples of the soiled bedding that were also used in the behavioural trials.

## RESULTS

### Preliminary controls

In total we detected 89 VOCs (Table S1) in the control and experimental samples. Seventy-two compounds that were present both in the experimental samples and in the controls were excluded from the analysis. The list of 17 compounds specific to the experimental samples is given in Table 1. We performed an RDA analysis on the results obtained with the preliminary study aimed to assess adsorption consistency between fibres and fibre wear effect on VOC detection. The model included the relative proportion of the 17 VOCs with ‘fibre identity’ (two modalities) and ‘sampling occasion’ (seven modalities) as factors (model 1, Table 2). While VOC composition did not vary with fibre identity (*F*=0.957, *P*=0.395), it did vary among sampling occasions (*F*=34.185, *P*<0.001) (model 1; Table 2). Variation between sampling occasions was not linear and hence may not relate to fibre wear (Fig. S1). Rather, although the same quantity of soiled bedding from the same odour source was analysed, variation in the mixture of different body fluids present in each aliquot (here 3 g), due to sampling of a heterogeneous source, might have been the cause of the variation between sampling occasions. Based on these considerations, three aliquots of each odour source were analysed in the main study, to take into account potential sampling bias when addressing the impact of cancer development on VOC composition (see below).

**Table 1.**
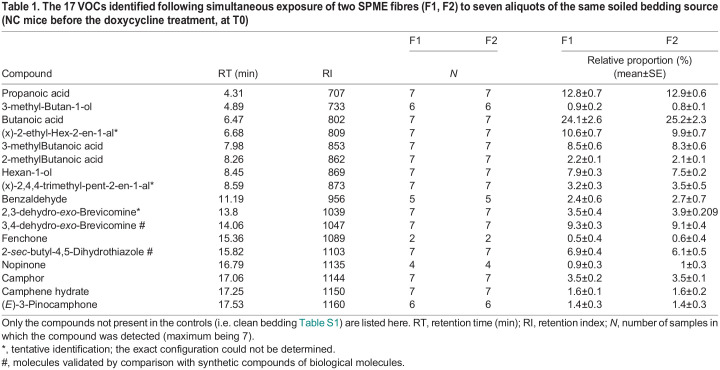
The 17 VOCs identified following simultaneous exposure of two SPME fibres (F1, F2) to seven aliquots of the same soiled bedding source (NC mice before the doxycycline treatment, at T0)

**Table 2.**
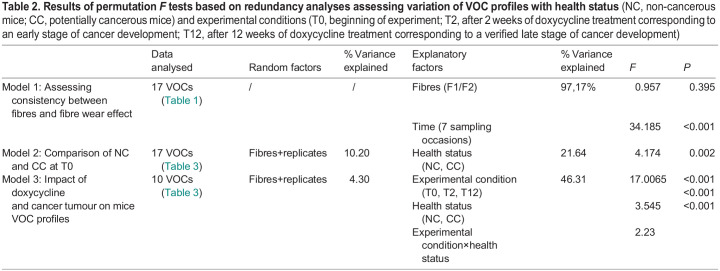
**Results of permutation *F* tests based on redundancy analyses assessing variation of VOC profiles with health status** (NC, non-cancerous mice; CC, potentially cancerous mice) and experimental conditions (T0, beginning of experiment; T2, after 2 weeks of doxycycline treatment corresponding to an early stage of cancer development; T12, after 12 weeks of doxycycline treatment corresponding to a verified late stage of cancer development)

### Cancer influence on VOC production

We did not detect qualitative differences between cancerous (CC) and non-cancerous (NC) mice VOC profiles, both comprised the same 17 compounds (Table 3). In the behavioural study (Gouzerh et al., 2022a), the wild-derived mice did not discriminate between soiled bedding of CC versus NC mice before the start of the doxycycline diet (T0), we hence expected that their VOCs would not differ. Contradicting our expectation, the GC/MS analysis indicated that the VOC profiles of CC and NC were significantly different at T0 (model 2; Table 2). Seven compounds that had an absolute correlation >0.8 with the RDA main axes were identified as being involved in the above-mentioned differences (Table 3). We hence considered these molecules not to be related to the mice discriminatory behaviour of CC versus NC. The next analysis considered only the ten VOCs that did not differ between CC and NC at T0 (model 3; Table 2). In this analysis the two random factors –replicate (three aliquots) and fibre identity – accounted for a negligible part of the total variance, respectively, 3.32% and 1.05%. Experimental conditions (T0, T2, or T12), health status (NC or CC), and their interaction were all found to be significant (*P*<0.005; Table 2). As expected with this new dataset, we did not detect a significant difference between CC and NC mice at T0 (*P*=0.622). Furthermore, wild-derived mice discriminated between soiled bedding of CC versus NC mice at T2, when they were habituated to a NC stimulus, but did not when they were habituated to a CC stimulus (Gouzerh et al., 2022a). We hence expected VOCs of CC and NC to differ at T2, which was not confirmed (*P*=0.495). Still, the levels of the ten VOCs at T2 differed significantly from those at T0 (*P*<0.001), indicating that the doxycycline treatment induced changes in the stimuli VOC profiles. Specifically, four of the ten VOCs analysed were affected by the doxycycline treatment (i.e. differences between T0 and T2 that were similar in both CC and NC): namely, the hexan-1-ol, 3-methyl-butan-1-ol, *(x)*-2,4,4-trimethyl-pent-2-enal, and fenchone. The hexan-1-ol and 3-methyl-butan-1-ol were either absent or present as a trace before the start of the doxycycline treatment (Table 3), while *(x)*-2,4,4-trimethyl-pent-2-enal disappeared from the VOCs profiles after the start of the treatment.

**Table 3.**
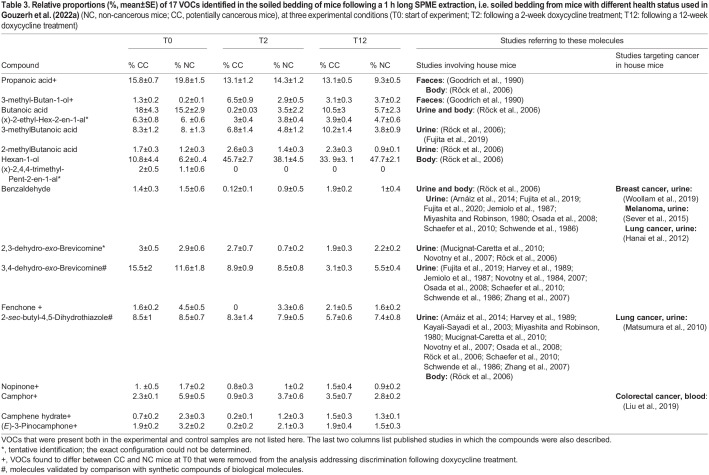
**Relative proportions (%, mean±SE) of 17 VOCs identified in the soiled bedding of mice following a 1 h long SPME extraction, i.e. soiled bedding from mice with different health status used in Gouzerh et al. (2022a)** (NC, non-cancerous mice; CC, potentially cancerous mice), at three experimental conditions (T0: start of experiment; T2: following a 2-week doxycycline treatment; T12: following a 12-week doxycycline treatment)

**Fig. 1.**
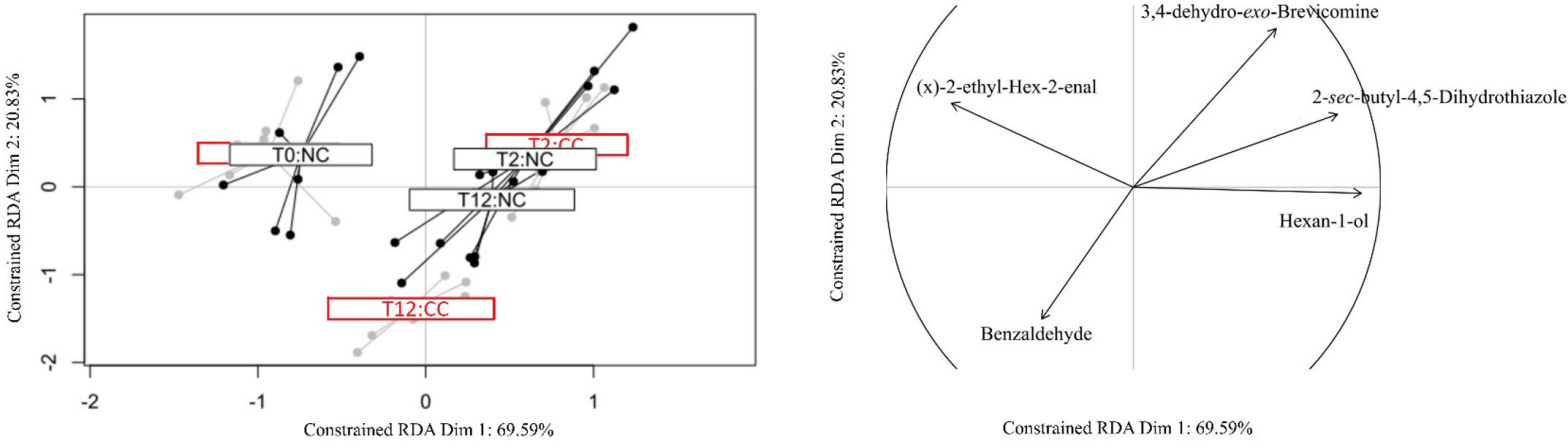
**Redundancy analysis (RDA) plots picturing, in the left panel, the outcome of model 3 (Table 2) testing the effect of health status (NC/CC) and experimental conditions (T0-T12) on variation of VOCs levels.** The correlation circle indicates compounds showing an absolute correlation coefficient >0.8 with the two main axes of the RDA.

The VOC profiles of NC and CC were significantly different at T12 (*P*<0.001), a pattern consistent with the behavioural results (Gouzerh et al., 2022a). Further, the doxycycline treatment did not induce a change in the VOC profile of NC mice between T2 and T12 (*P*=0.259), while the CC profile at T12 was significantly different from that at T2 and from that of NC at both T2 and T12, indicating that lung adenocarcinoma influenced the VOC profiles of CC mice at T12 when the tumour was fully developed (Fig. 1). Moreover, here too four out of the ten VOCs analysed differed between the profiles of CC mice at T2 and T12. Three of these compounds, 3,4-dehydro-*exo*-brevicomine, 2-sec-butyl-4,5-dihydrothiazole, and hexan-1-ol, were present in lower relative proportions in CC mice at T12 compared to both NC and CC individuals at T2. The fourth compound, benzaldehyde, was present in higher relative proportions in CC at T12 (Table 3).

## DISCUSSION

In an earlier study, we highlighted the ability of mice to identify the odour of soiled bedding of a cancerous congener at a very early stage of cancer development (Gouzerh et al., 2022a). Olfactory cues are used by mice in their everyday social and sexual communication (Asaba et al., 2014; Hurst, 2005). Potential candidates involved in the behavioural discrimination evidenced in our earlier study (Gouzerh et al., 2022a) are VOCs as well as non-volatile compounds such as proteins (MUPs), peptides (Kimoto et al., 2005; Roberts et al., 2010) or sulphated steroids (Fu et al., 2015). Many studies have pointed out that cancer could induce body odour changes involving VOCs (Beger, 2013; Gouzerh et al., 2022b; Krilaviciute et al., 2015). We therefore made the hypothesis that VOCs could be involved in the behavioural discrimination of soiled bedding of CC evidenced in our earlier study. The results of the present study indicate that, although differences in some VOC compounds present in soiled bedding of CC as compared to NC could explain the behavioural discrimination at a late stage of cancer development (T12), we did not identify the molecules that could explain behavioural discrimination of stimuli collected at an early stage of tumour development (T2). These findings suggest that either the mice involved in the behavioural trials could detect VOC differences that the GC/MS approach was not sensitive enough to detect, or that other type of molecules (e.g. proteins, peptides, etc.) present in the soiled bedding, not detectable by GC/MS, may (also) be involved.

We identified 17 VOCs in the soiled bedding that was used as the odour source in our behavioural study, among which seven were not reported previously in studies analysing mice VOCs (Table 3). The mice did not behaviourally discriminate between soiled bedding of the two lines before the doxycycline treatment at T0 (Gouzerh et al., 2022a); however, in the present study we identified significant quantitative differences in the VOC profiles of the two lines at T0, suggesting that these differences may not include informative discriminatory cues. The two mice lines were kept under exactly the same conditions and were litter mates of the same age. Hence, differences in their VOC profiles at T0 most probably relate to relatively small differences in their genetic backgrounds, i.e. the presence versus the lack of CCSP-rtTA and EGFRT790M/L858R. Small differences in genetic background are known to induce changes in mice odour characteristics (Schaefer et al., 2002; Yamazaki et al., 1990). Moreover, cyclic enol ethers, ketone, dehydrobrevicomin, and thiazoline were shown to be present in different concentrations in urine of mice differing by a haplotype of the t complex genotype (Jemiolo et al., 1991). In the present study seven compounds differed between NC and CC at T0. Except for 3-methyl-butan-1-ol, all compounds were present in greater relative proportion in NC than in CC mice, suggesting that the presence of CCSP-rtTA and EGFR^T790M/L858R^ in the CC line may induce a reduction in the production of these VOCs. Among the seven compounds found to differ between the two mouse lines, only propanoic acid and 3-methyl-butan-1-ol were reported in the mouse VOC literature (Goodrich et al., 1990); the other five compounds – fenchone, nopinone, camphor, camphene hydrate, and (E)-3-pinocamphone – were terpenes also found in plants and only camphor was reported as a mouse VOC in a study that analysed the blood of mice with colorectal cancer (Wang et al., 2019).

We did not find a significant difference between the VOC profiles of CC and NC soiled bedding at T2, although wild-derived mice discriminated between the two soiled bedding types. As mentioned earlier, discrepancies between the behavioural and chemical results suggest that either other odorant molecules than VOCs were (also) involved in behavioural discrimination of mice at T2, and/or our method, SPME coupled with GC/MS, did not detect VOCs that the mouse nose could detect. Finally, consistent with the behavioural results, a difference between the VOC profiles of CC and NC was detected after 12 weeks of treatment with doxycycline (T12), when all mice had developed lung adenocarcinoma (Mur et al., 2020), suggesting that VOCs could be involved at least partially in the behavioural discrimination process evidenced earlier (Gouzerh et al., 2022a). Specifically, we identified four VOCs that were involved in these differences. Benzaldehyde was found in significantly higher proportions in CC than NC mice, a molecule also found in almonds and other plants, but whose metabolism is not yet understood.

This compound is commonly found in mouse urine (Arnáiz et al., 2014; Fujita et al., 2020; Kwak et al., 2008; Röck et al., 2006) and has been reported as a cancer biomarker in other cancer studies using mice (Hanai et al., 2012; Sever et al., 2015; Woollam et al., 2019). Benzaldehyde has also been found to be related to human cancers and might be considered as a relatively general biomarker of lung (Barash et al., 2012; Davies et al., 2014; Jia et al., 2018; Ligor et al., 2009), colorectal (Altomare et al., 2020), and breast (Silva et al., 2017) cancers. Although this compound was described mostly in cell cultures and was found in higher concentrations in cancerous as compared to non-cancerous cells, here we report its emission in mice body fluids. Benzaldehyde was also shown to have an anti-tumour action in some cases (Ariyoshi-Kishino et al., 2010; Kochi et al., 1980; Saitoh and Saya, 2016), which may seem contradictory with its high concentration in CC mice (this study) and in tumoral cells (Ariyoshi-Kishino et al., 2010; Saitoh and Saya, 2016). The three other candidate VOCs identified in this study as potentially explaining the behavioural discrimination between CC and NC mice soiled bedding at T12 were found in smaller proportions in CC compared to NC mice, namely, hexan-1-ol, 2-*sec*-butyl-4,5-dihydrothiazole (thiazoline), and 3,4-dehydro-*exo*-brevicomine (brevicomine). Among these compounds, brevicomine and thiazoline are known male mouse pheromones, and were shown to be involved in male dominance and attractiveness to female mice (Novotny et al., 2003). Thiazoline was also proposed as a candidate biomarker for lung cancer in another mouse study (Matsumura et al., 2010). Thus, a cancerous tumour might affect pheromone emissions in sick mice and could impact their social life. However, the lower levels of the two major male pheromones in cancerous mice soiled bedding did not seem to influence female preference in trials presenting NC and CC stimuli at a late stage of tumour development, further suggesting that the changes may be too small to induce a significant effect on female behaviour. The third candidate molecule, hexan-1-ol, was described in urine of healthy mice (Röck et al., 2006). In our study, hexan-1-ol was found in smaller proportions in CC compared to NC individuals, consistent with other investigations of human melanoma (Kwak et al., 2013) and head and neck cancers (Shigeyama et al., 2019).

Soiled bedding contains VOCs emitted by mice during routine activities in their cages, making its sampling non-invasive and non-stressful for the mice. It also contains VOCs from a variety of sources, e.g. faeces, urine, saliva, preputial gland. Nevertheless, VOCs are not the only odorant molecules present in soiled bedding. Discrepancies between the earlier behavioural study (Gouzerh et al., 2022a) and the present chemical analysis of VOCs present in the soiled bedding involved in the behavioural trials strongly suggest that some of the molecules identified here (at T0) are not effective odorant signals, and that other odorant molecules, supposedly proteins, peptides or other, might be involved in behavioural discrimination of cancerous stimuli at an early stage of tumour development (T2). Finally, our study identified four VOCs that differed significantly between soiled bedding of CC and NC mice at a late stage of tumour development. These molecules could be odorant signals involved in behavioural discrimination of CC and NC stimuli. Further investigations involving bioassay presenting the four candidate molecules as stimuli to mice nose should help validate/invalidate their role in behavioural discrimination.

## MATERIALS AND METHODS

### Ethical clearance

Our study received ethical clearance from the Ethical Committee for Animal Experimentation (French Ministry of Higher Education, Research and Innovation, number 1645-22123). All the recommendations for animal welfare were followed during our experiments.

### Animals

Two genotypes were involved in this study: WT and CCSP-rtTA/EGFR^T790M/L858R^ (Li et al., 2007; Mur et al., 2020). WT mice (hereafter referred to as non-cancerous or NC), lacking the two transgenes, CCSP-rtTA and EGFR^T790M/L858R^, do not develop tumours upon doxycycline (antibiotic) induction. CCSP-rtTA / EGFR^T790M/L858R^ mice, which carry the two transgenes, develop lung adenocarcinoma upon doxycycline induction; these are referred to as CC mice. To induce lung specific tumour development mice received food supplemented with doxycycline (1 mg/kg). The treatment lasted 12 weeks and started when the mice were 13 weeks old.

NC and CC male mice were obtained from the Montpellier Cancer Research Institute (IRCM) at the age of 6 weeks and maintained at the Institute of Research and Development breeding facilities in Montpellier. Before the experiment began, mice were maintained in groups of two to four mice in transparent plastic cages (26.8 cm W×21.5 cm L×14.1 cm H). Each cage contained sawdust, a cellulose square, hay, and a cardboard tunnel. The mice were observed and weighed once a week throughout the experimental period to monitor their health. All mice were euthanized at the end of the experiment (at 25 weeks of age). The non-cancerous or the cancerous status of mice was confirmed by histopathological analysis of Haematoxylin and Eosin stain of whole lung sections prepared after necropsy.

### Odorant stimuli collection and experimental protocol

**Fig. 2.**
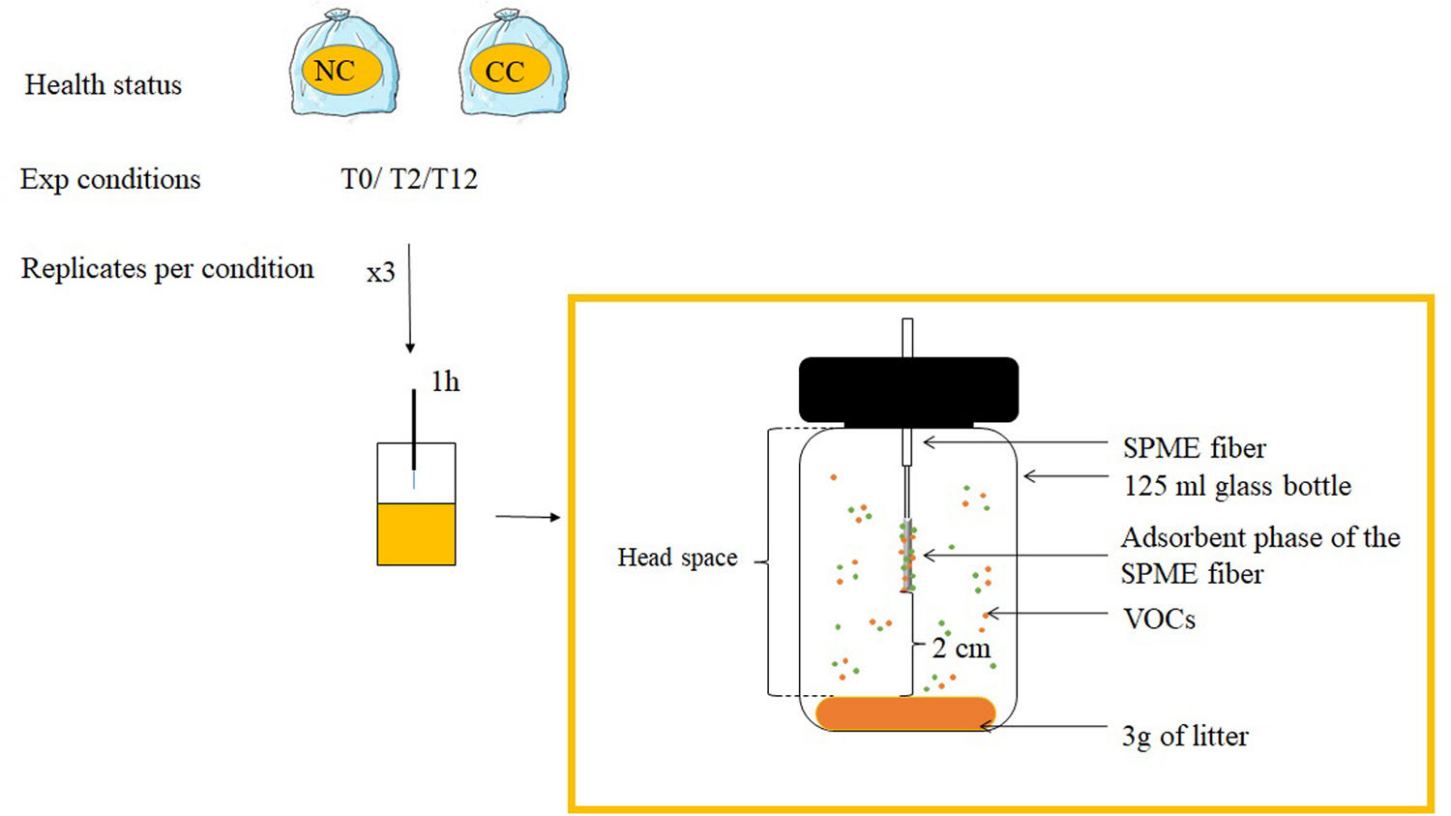
**Schematic representation of the experimental protocol.** Each bag contained pooled soiled bedding from three to four different mice. These pools of soiled bedding were also used as stimuli in the earlier behavioural study (Gouzerh et al., 2022a). Soiled bedding was sampled at different times (experimental conditions): T0: before treatment; T2: after 2 weeks of doxycycline treatment; T12: after 12 weeks of doxycycline treatment. The adsorbent phase of the SPME fibre was exposed for 1 h to a given soiled bedding sample.

Stimuli were 2-week-old soiled bedding of CC and NC mice. Each mouse was isolated at the age of 10 weeks in a cage (26.8 cm W×21.5 cm L×14.1 cm H) containing 130 g sawdust and a cellulose square. The housing conditions of all mice were as homogenised as possible, and all mice were given the same quantity of food. Every 2 weeks, the entire volume of soiled bedding from each cage was collected in a plastic bag, kept at −20°C, and replaced by 130 g of clean sawdust. Soiled bedding was collected from the cages of three to four mice housed individually and mixed for the purpose of the behavioural study (Gouzerh et al., 2022a). In this study we analysed the VOC composition of the same samples. These odour sources were obtained at three experimental conditions: T0, before the start of doxycycline treatment; T2, after 2 weeks of doxycycline treatment (early stage of cancer tumour development); and T12, after 12 weeks of doxycycline treatment (Fig. 2).

Altogether, 18 odour sources were involved in the earlier behavioural study (Gouzerh et al., 2022a) and analysed here. Each of the 18 sources was processed in triplicate to control for sampling bias, i.e. heterogeneity inherent to the rough structure of soiled bedding: an uneven mixture of several body fluids in the sawdust. In total, we analysed 54 experimental samples along with ten samples of clean bedding (one per day) to serve as controls during the 10 days of VOC extraction and chemical analyses. Solid-phase micro-extraction (SPME), the most common method to extract VOCs (Chen and Pawliszyn, 1995; Deng and Zhang, 2004) was used in this study. Two PMDS-DVB SPME fibres were used alternatively in this part of the study, randomizing their use among NC and CC.

Because we used two different SPME fibres in our study, in a preliminary test we assessed consistency of VOC adsorption by exposing the two fibres simultaneously to the same stimuli. To this aim we compared the VOC profiles of seven aliquots of the same NC soiled bedding (at T0) on seven different occasions, which led us to also address the influence of fibre wearing on VOCs extraction.

### VOC extraction

We used PMDS-DVB (polydimethylsiloxane and divinylbenzene) SPME fibres (65 μm thickness; Stable Flex 24Ga, Sigma-Aldrich, Bellefonte, PA, USA). PMDS-DVB is the most polyvalent type of SPME fibre, which is relevant when molecules with different chemical characteristics are extracted as was the case here. The experimental samples were processed as follows. A frozen soiled bedding bag was thawed on ice during the entire procedure to avoid evaporation. A 3 g aliquot was transferred into a 125 ml glass vial that was then sealed with a rubber septum. The vial was placed in an oven maintained at 22°C during 3 min. The SPME fibre was introduced into the vial after piercing the septum with a needle and was always positioned ∼2 cm above the odour source (Fig. 2). The PMDS-DVB phase was then exposed to the head space of the vial for 60 min.

### VOC characterisation by gas chromatography - mass spectrometry (GC-MS)

The SPME extract was injected into a quadrupole QP2010-SE GC-MS (Shimadzu, Kyoto, Japan) for identification and relative quantification of its chemical content. Desorption was achieved by inserting the PMDS-DVB phase of the SPME fibre into the ultra-inlet liner (for SPME/purge and trap, 0.75 mm ID; Agilent CrossLab, Santa Clara, CA, USA) placed in split/splitless GC-MS injector heated to 250°C. The injection lasted three minutes and was made using a split ratio of 1:4 to allow a Gaussian form of low boiling point compounds, and hence a better separation on the chromatogram and further integration. The GC was equipped with an Optima 5-MS fused silica capillary column (30 m×0.25 mm×0.25 µm film thickness; Macherey-Nagel, Düren, Germany). Helium was used as the carrier gas (1 ml min^−1^). The GC temperature was maintained at 40°C for 2 min, after which the temperature increased by 5°C every minute until it reached 175°C, and then by 12°C min^−1^ until it reached 220°C.

### Identification of VOCs present in the extracts

Spectra were analysed with the software GCMS Solution (Shimadzu, Kyoto, Japan). Retention indices (RI) were calculated using as a reference the retention time of a series of n-alkanes injected directly in the GC-MS (Alkanes standard solution, 04070, Sigma-Aldrich). Compounds were identified by spectral analysis (NIST 2011, Wiley 292 Registry Ninth), RI comparisons with reference databases, (e.g. Adams, 2007, Pubchem, https://pubchem.ncbi.nlm.nih.gov/), and comparison with injected synthetic compounds of biological molecules, when available, to confirm their presence in the VOCs’ profile. Based on the total ion current chromatogram (TICC), the peak surface area of each compound was calculated, and its value was used for further comparisons between samples (see below). To be conservative, we excluded compounds that were present in both our controls (clean bedding) and our experimental samples (soiled bedding) from the analyses. The peak surfaces of compounds identified by their mass spectra but only present in trace amounts could not be calculated and were given an arbitrary value corresponding to 10% of the surface area of the smallest peak identified in the entire dataset. Because the peak surface area of a compound obtained with the SPME method (adsorption) is a relative quantity varying with the number and quantity of all the other compounds present in the VOCs profile, to compare different profiles we considered the relative proportion of each compound in a given chromatogram/profile. To this aim, the peak areas for all compounds were summed for each chromatogram, and their relative proportions were calculated as the ratio of a given compound's surface area divided by the sum of the surface areas of all the compounds identified in that chromatogram.

### Statistical analyses

All statistical analyses were performed with Rstudio version 3.4.4 (R Core Team, 2020). We used the following packages: ade4 (Thioulouse et al., 2018), vegan (Oksanen et al., 2020), mixOmics (Rohart et al., 2017), ggplot2 (Wickham, 2014), RVAideMemoire (Hervé, 2022), and Hotelling (Curran, 2021). Variations in the relative proportions of VOCs were assessed with a multivariate approach, i.e. redundancy analysis (RDA) followed by permutation *F* tests (Hervé et al., 2018) using the R package vegan (Oksanen et al., 2020). Relative proportions were CLR-transformed prior to each RDA. Because our data included zeroes, a small constant, an order of magnitude smaller than the smallest non-zero value in the data, was added to all values prior to transformation (e.g. 0.01 if the smallest non-zero value was 0.1). Data were then autoscaled to give equal weight to all compounds in the analyses.

An RDA analysis used to assess consistency in VOCs extraction by the two fibres exposed simultaneously to a standard stimulus in our preliminary study. Model 1 included fibre identity (F1 or F2) and sampling occasion (7 modalities) as fixed factors.

The main study data set (56 samples corresponding to three aliquots of each of the 18 odour sources) obtained after a 1 h extraction was also analysed using RDA models to identify impact of cancer on VOCs composition (Model 2, Table 2). We sought to determine whether the VOCs composition of NC and CC odour sources at T0 were similar, so we included health status (NC, CC) as a fixed factor with replicates (three aliquots per sample) and fibre identity (two fibres used) as random factors (‘condition’ in the RDA). Model 2 excluded the VOCs identified as different between NC and CC before the doxycycline treatment (at T0) and addressed whether the rest of the VOCs differed according to health status, experimental conditions (T0, T2, T12), and their interactions (with replicate and fibre identity as random factors). When relevant, for a given model, we identified and listed the VOCs showing an absolute correlation coefficient higher than 0.8 with the main constrained axes of the RDA.

## Supplementary Material

10.1242/biolopen.060324_sup1 Supplementary information
