# Supplementary material for: Partial role of volatile organic compounds in behavioural responses of mice to bedding from cancer-affected congeners
Source: Biol Open. 2024 Oct 1;13(10):bio060324. doi: 10.1242/bio.060324 (PMC11552615; doi:10.1242/bio.060324)
Supplement: Supplementary information [file biolopen-13-060324-s1.pdf]

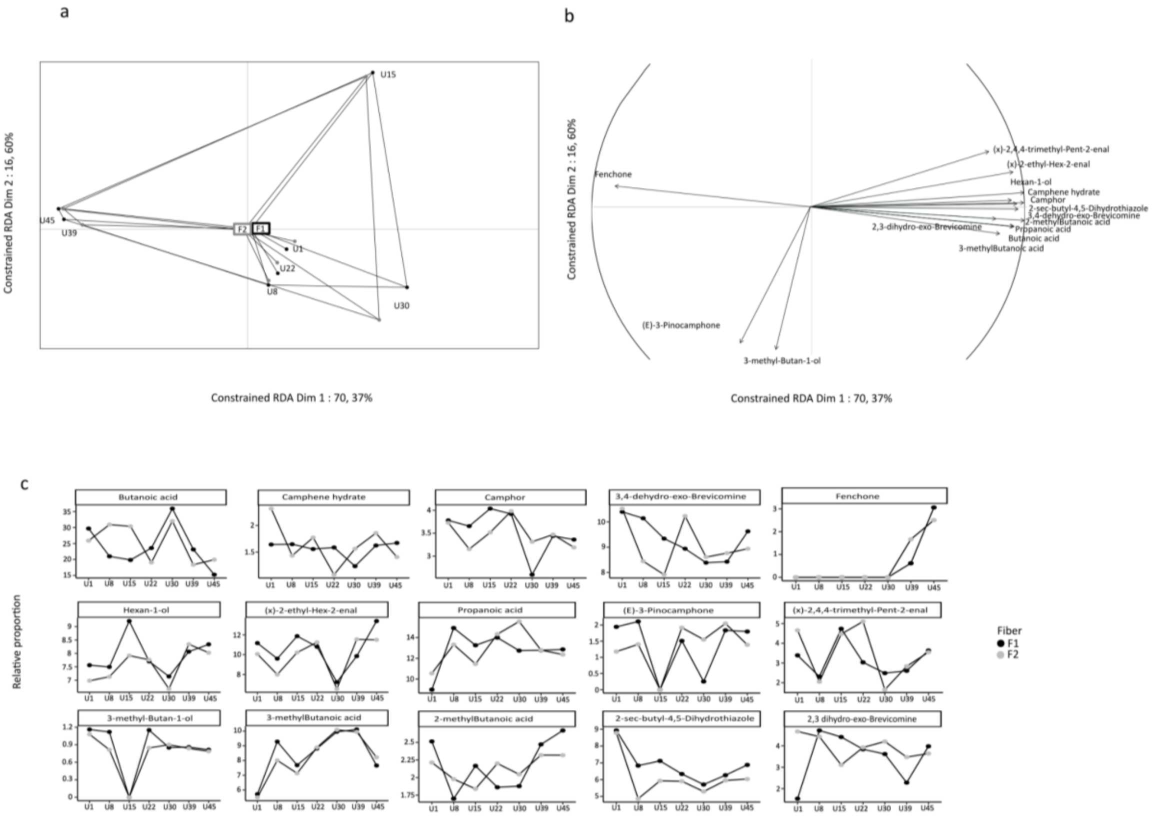

Fig. S1. Results of the preliminary study assessing consistency between fibers and fiber wear effect on the VOCs profiles. a) Score plots results of the redundancy analysis (RDA) comparing variations in the relative proportions of 17 volatile organic components (VOCs) extracted by two fibers simultaneously at seven different occasions (order of use varying from 1 (U1) to 45 (U45)); b) The correlation circle identifies VOCs showing an absolute correlation coefficient > 0.8 with the two main c) Patterns of variation of the VOCs identified above (box plots include median, minimum and maximum, and upper and lower quartile values).

Table S1. Complete l**ist of volatile organic components identified in the experimental samples and the controls.** In blue: compounds found only in soiled bedding and retained for our analyses; In pink: the n-alkanes used to calculate compound retention indices (RI). RT: retention time (min); NI: compounds not identified.

Compound	RT	RI
n-Hexane	2.79	600
Acetic acid	2.83	603
Pentanal	4.05	692
n-Heptane	4.16	700
Propanoic acid	4.31	707
3-methyl-Butan-1-ol	4.89	733
NI	4.98	737
Toluene	5.55	762
Pentan-1-ol	5.62	765
Hexanal	6.35	798
n-Octane	6.40	800
Butanoic acid	6.47	802
2-ethyl-Hex-2-enal	6.68	809
3-methylButanoic acid	7.98	853
ethylBenzene	8.12	858
2-methylButanoic acid	8.26	862
nXylene	8.38	866
Hexan-1-ol	8.45	869
(x)-2,4,4-trimethyl-Pent-2-enal	8.59	873
NI	8.90	884
Styrene	9.00	887
Pentanoic acid	9.19	893
Heptanal	9.30	897
n-Nonane	9.39	900
NI	9.50	903
α-Pinene	10.48	934
Hept-2-enal	11.06	952
Benzaldehyde	11.19	956
Heptan-1-ol	11.60	969
β-Pinene	11.84	977
Oct-1-en-3-ol	11.88	978
6-methyl-Hept-5-en-2-one	12.03	983
2-pentylFuran	12.23	989
Hexanoic acid	12.44	996
Octanal	12.53	998
n-Decane	12.58	1000
δ-3-Carene	12.90	1010
p-Cymene	13.32	1024
Limonene	13.48	1029
NI	13.65	1034
2,3-dihydro-*exo*-Brevicomine	13.80	1039
6-methyl-Heptan-2-one	13.96	1044

Biology Open • Supplementary information

3,4-dehydro-*exo*-Brevicomine	14.06	1047
Oct-2-enal	14.28	1054
vinylHexanoate<br>Unknown compound 1	14.69	1067
Octan-1-ol	14.80	1071
Heptanoic acid	15.19	1083
Fenchone	15.36	1089
Cymenene	15.34	1088
n- Undecane	15.72	1100
Nonanal	15.73	1100
2-*sec*-butyl-4,5-Dihydrothiazole	15.82	1103
Fenchol	16.18	1115
NI	16.60	1129
Nopinone	16.79	1135
'(*E*)-Pinocarveol	16.95	1140
Camphor	17.06	1144
(*Z*)-Verbenol	17.13	1146
Camphene hydrate	17.25	1150
(*E*)-Non-2-enal	17.45	1157
(*E*)-3-Pinocamphone	17.53	1160
Pinocarvone	17.60	1162
Borneol	17.79	1168
(*Z*)-3-Pinocamphone	17.96	1174
4-Terpineol	18.12	1179
NI	18.19	1181
p-Cymen-8-ol	18.27	1184
α-Terpineol	18.37	1187
Unknown Monoterpene derivative 1	18.50	1191
Myrtenal	18.60	1195
Myrtenol	18.70	1198
n-Dodecane	18.76	1200
Decanal	18.80	1201
Verbenone	18.98	1208
2,4-Nonadienal	19.05	1210
Unknown compound 2	19.14	1213
Benzothiazole	19.40	1223
NI	19.63	1231
Cumin aldehyde	19.85	1238
Carvone	19.96	1242
Piperitone	20.27	1253
(*E*)-Dec-2-enal	20.43	1259
Undecan-2-one	21.31	1290
1-methyl-Naphthalene	21.43	1294

Biology Open • Supplementary information

N,N-dibutyl-Formamide	21.60	1300
n-Tridecane	21.60	1300
NI	22.75	1343
α-Terpinyl acetate	22.94	1350
NI	23.22	1361
4-*tert*-butyl-Cyclohexyl acetate	23.53	1372
NI	23.76	1381
n-Tetradecane	24.27	1400
NI	24.57	1412
Geranyl acetone	25.53	1450
n-Pentadecane	26.81	1500
(*E*)-Nerolidol	28.38	1572
n-Hexadecane	29.00	1600
Caryophyllene oxide	29.01	1600
Sesquiterpenol Inconnu	29.89	1641
(6*Z*,9*E*)-Heptadeca-6,9-diene	30.60	1673
n-Heptadecane	31.13	1700
